# Familial Associations of Colorectal Cancer with Other Cancers

**DOI:** 10.1038/s41598-017-05732-z

**Published:** 2017-07-12

**Authors:** Hongyao Yu, Akseli Hemminki, Kristina Sundquist, Kari Hemminki

**Affiliations:** 1 Division of Molecular Genetic Epidemiology, German Cancer Research Center (DKFZ), Im Neuenheimer Feld 580, D-69120 Heidelberg, Germany; 2 Cancer Gene Therapy Group, Faculty of Medicine, University of Helsinki, Helsinki, Finland; 3 Helsinki University Hospital Comprehensive Cancer Center, Helsinki, Finland; 4 Center for Primary Health Care Research, Lund University, 205 02 Malmö, Sweden

## Abstract

Colorectal cancer (CRC) has a strong familial component which extends to discordant cancers (ie non-CRC tumors). This is best seen in cancer syndromes such as hereditary non-polyposis colorectal cancer (HNPCC) which predisposes to several tumor types. Population-based family studies have also found discordant associations for CRC but they have included cancers which manifest in HNPCC, and there is no convincing evidence of discordant associations beyond the known syndromes. We address familial associations of non-CRC tumors with CRC using the resources of the Swedish Family-Cancer Database and applying a powerful approach of assessing familial relative risks in families of increasing numbers of patients with discordant cancers. Among 1.8 million cancer patients and over 200,000 CRC cases consistent familial associations of CRC was observed for several HNPCC related cancers. However, for small intestinal, pancreatic and nervous system cancers RRs remained essentially unchanged when potential HNPCC families were excluded, suggesting involvement of genes not related to HNPCC. Two independent associations of CRC were found for melanoma, thyroid and eye cancers and these appeared not to be related to known syndromes. A number of other cancers associated with CRC in single analyses and independent studies are required to assess the relevance of such findings.

## Introduction

About 13% of patients with colorectal cancer (CRC) have a parent or a sibling diagnosed with CRC and the familial relative risks (RRs) are 1.80 and 2.00, respectively^1, 2^. Risks between spouses have been less than 1.10, suggesting that familial clustering can mostly be explained by genetic susceptibility. Hereditary non-polyposis colorectal cancer (HNPCC) is the most common CRC syndrome, accounting for some 10% of familial CRC^3, 4^ (for distinction between HNPCC and Lynch syndrome see Methods). Exome sequencing of 625 early-onset familial CRC cases found deleterious mutations in 89 (14%) patients, mismatch repair gene mutations being the most common (76.4%), followed by *APC* (11.2%), *MUTYH* (7.9%) and *POLE and POLD1* (3.4%)^3^. However, variants of uncertain relevance may increase this figure if they are found to be deleterious^3, 5^. Additionally, numerous (at least 50) low-risk susceptibility loci have been identified^6^. However, as their RRs are low they combined contribute to the familial risk no more than the high-risk genes^7^. Thus, known genes appear to explain less than 30% of the empirical familial risk of CRC.

HNPCC, familial adenomatous polyposis (FAP) and other rare CRC-related cancer syndromes manifest also cancers other than CRC; in HNPCC the risk of at least 8 cancers is increased^4, 8, 9^. Also population-based family studies have shown shared association of CRC and a few other cancers, such as endometrial and pancreatic cancers^10, 11^. Data for associated discordant (i.e., other) cancers may provide clues about shared genetic pathways or environmental risk factors of these cancer and CRC.

We use here stringent statistical criteria to search for discordant familial associations with CRC and other cancers using the most recent update of the Swedish Family-Cancer Database, the largest family dataset in the world^12^. The approach involves a two-way comparison, i.e., assessment of familial RRs for cancer X in families with increasing numbers of patients with CRC, or conversely, familial RRs for CRC in families with increasing numbers of patients with cancer X. As HNPCC manifests many different cancers we wanted to test if discordant associations are found if the number if HNPCC families were reduced, by removing any families presenting double primaries of typical HNPCC related cancers^9, 13^.

## Results

The nationwide Swedish Family-Cancer Database includes 4.3 million families and 1.8 million cancers reported to the national Cancer Registry. A total of 207,512 individuals were diagnosed with CRC, and of these 35,360 were in the 0–80 year old offspring generation used as index individuals to calculate RRs. Exclusion of potential HNPCC families (see Methods), removed 16,160 (0.37%) families, including 41,201 individuals without cancer and 22,656 cancer patients.

In Table 1 all cancers are shown which were significantly associated with CRC in any of two-way comparisons (even considering the trend test), including 14 discordant cancers. As reference we show RRs for concordant CRC which increased from 1.76 (1 proband diagnosed with CRC) to 5.13 (at least 3 probands diagnosed with CRC). All RRs were significant at 0.1% confidence levels. Considering significant trend tests for both of the two-way analyses, small intestinal, pancreatic, endometrial, ovarian and nervous system cancers showed consistent increases. However, with the exception of endometrial cancers, almost all significant associations were limited to families of one affected proband. For thyroid cancer, 3 RRs were increase, for eye cancer 2 RRs were increased and for the remaining cancers only one RR was increased. The exception was stomach cancers for which only one trend test was increased. The mean age at diagnosis for CRC in thyroid cancer families was 59.3 years compared to 58.7 years in all CRC; the 5 CRCs with an RR of 4.28 (2 thyroid cancers in probands) were diagnosed at the mean age of 61.4 years. We looked in more detail on eye cancer histology: half of the cases were ocular melanomas but none of the RRs for this histological type reached statistical significance.

**Table 1:** Discordant and concordant risks for CRC.

**Risk in offspring**	**Proband cancer**	**1 cancer case in the family**			**2 cancer cases in the family**			**>=3 cancer cases in the family**			**Trend test**
		**Cases**	**RR** ^1^	**95% CI** ^2^	**Cases**	**RR**	**95% CI**	**Cases**	**RR**	**95% CI**	**P-value**
Stomach	Colorectum	479	1.09	(0.99–1.21)	29	1.38	(0.93–2.05)	3	2.56	(0.76–8.64)	**0.0269** ^6^
Colorectum	Stomach	1178	1.06	(0.81–1.37)	23	1.35	(0.22–8.36)	0	.	.	0.6672
Small intestine	Colorectum	185	**1.28** ^4^	(1.10–1.49)	11	1.65	(0.90–3.00)	2	**6.00** ^3^	(1.48–24.33)	**0.0007**
Colorectum	Small intestine	160	**1.33**	(1.11–1.59)	0	.	.	0	.	.	**0.0026**
Colorectum	Colorectum	4786	***1.76*** ^5^	(1.70–1.82)	381	***2.93***	(2.60–3.30)	36	***5.13***	(3.48–7.54)	** < 0.0001**
Pancreas	Colorectum	612	**1.13**	(1.03–1.25)	35	1.33	(0.90–1.97)	3	1.96	(0.52–7.49)	**0.0040**
Colorectum	Pancreas	861	***1.15***	(1.07–1.23)	13	1.52	(0.87–2.63)	0	.	.	**<0.0001**
Lung	Colorectum	2159	1.07	(1.00–1.14)	101	1.03	(0.77–1.37)	4	0.71	(0.17–3.01)	0.0621
Colorectum	Lung	1972	**1.07**	(1.02–1.13)	70	1.09	(0.84–1.42)	3	1.02	(0.29–3.61)	**0.0082**
Breast	Colorectum	6507	**1.06**	(1.01–1.10)	300	1.08	(0.89–1.31)	11	0.82	(0.29–2.27)	**0.0158**
Colorectum	Breast	3278	1.03	(0.99–1.08)	185	1.09	(0.93–1.29)	5	0.52	(0.19–1.42)	0.0999
Endometrium	Colorectum	1025	1.09	(1.00–1.19)	68	**1.47**	(1.07–2.03)	4	1.62	(0.43–6.07)	**0.0098**
Colorectum	Endometrium	912	***1.21***	(1.12–1.32)	21	**2.22**	(1.32–3.73)	0	.	.	**<0.0001**
Ovary	Colorectum	861	**1.10**	(1.02–1.19)	40	1.12	(0.78–1.60)	4	2.15	(0.70–6.64)	**0.0237**
Colorectum	Ovary	685	**1.09**	(1.01–1.18)	6	1.00	(0.44–2.27)	0	.	.	**0.0252**
Prostate	Colorectum	5451	1.02	(0.99–1.05)	253	0.99	(0.86–1.14)	13	1.01	(0.54–1.90)	0.3625
Colorectum	Prostate	3887	**1.04**	(1.01–1.08)	266	1.12	(0.98–1.28)	16	0.65	(0.38–1.12)	**0.0176**
Melanoma	Colorectum	2249	1.06	(1.00–1.12)	117	**1.30**	(1.02–1.66)	5	1.23	(0.38–4.04)	**0.0149**
Colorectum	Melanoma	839	1.04	(0.96–1.12)	28	1.50	(0.99–2.28)	2	3.40	(0.71–16.28)	0.1458
Eye	Colorectum	136	**1.20**	(1.01–1.41)	10	**2.11**	(1.17–3.79)	1	4.39	(0.69–27.74)	**0.0050**
Colorectum	Eye	76	0.94	(0.70–1.25)	0	.	.	0	.	.	0.6596
Nervous system	Colorectum	1568	**1.09**	(1.03–1.15)	71	1.19	(0.93–1.52)	6	2.10	(0.90–4.89)	**0.0011**
Colorectum	Nervous system	885	***1.14***	(1.06–1.24)	8	0.74	(0.33–1.65)	1	4.25	(0.44–41.53)	**0.0041**
Thyroid gland	Colorectum	442	**1.16**	(1.05–1.28)	24	**1.54**	(1.02–2.33)	0	.	.	**0.0009**
Colorectum	Thyroid gland	226	1.05	(0.93–1.19)	5	***4.28***	(1.85–9.91)	0	.	.	0.2262
Myeloma	Colorectum	393	**1.17**	(1.06–1.29)	23	1.44	(0.98–2.14)	1	1.21	(0.18–7.92)	**0.0006**
Colorectum	Myeloma	427	1.06	(0.96–1.17)	3	0.93	(0.29–2.99)	0	.	.	0.2603
CUP^7^	Colorectum	747	1.01	(0.88–1.15)	52	1.46	(0.89–2.39)	2	0.97	(0.08–12.08)	0.5309
Colorectum	CUP	1012	**1.14**	(1.05–1.24)	13	1.46	(0.69–3.09)	0	.	.	**0.0027**

^1^RR = relative risk.

^2^CI = confidence interval.

^3^Bold type denotes significantly increased RR at the two-sided 5% level.

^4^Bold and underlined value denotes significantly increased RR at the two-sided 1% level.

^5^Bold, underlined and Italics value denotes significantly increased RR at the two-sided 0.1% level.

^6^Bold type denotes that trend test was statistically significant.

^7^CUP = cancer of unknown primary.

Table 2 shows data for CRC associations when double primaries of potential HNPCC related cancers were removed. RRs for concordant CRC remained highly significant although they were decreased compared to Table 1. For endometrial and ovarian cancer almost all significant associations disappeared. For stomach cancer the trend test was no longer significant and for prostate cancer the single significant RR lost its significance, even though the magnitudes of RRs did not essentially change. For the other cancers RRs remained essentially unchanged between Tables 1 and 2, and for melanoma two RRs were significant compared to one RR in Table 1.

**Table 2:** Discordant and concordant risks for CRC in non-HNPCC families.

**Risk in offspring**	**Proband cancer**	**1 cancer case in the family**			**2 cancer cases in the family**			**>=3 cancer cases in the family**			**Trend test**
		**Cases**	**RR** ^1^	**95% CI** ^2^	**Cases**	**RR**	**95% CI**	**Cases**	**RR**	**95% CI**	**P-value**
Stomach	Colorectum	451	1.09	(0.98–1.21)	22	1.20	(0.76–1.90)	3	3.39	(0.98–11.70)	0.0644
Colorectum	Stomach	1092	1.05	(0.80–1.38)	23	1.45	(0.23–9.11)	0	.	.	0.6927
Small intestine	Colorectum	167	**1.25** ^4^	(1.06–1.47)	8	1.40	(0.68–2.87)	2	**8.23** ^3^	(1.96–34.47)	**0.0065** ^6^
Colorectum	Small intestine	143	**1.31**	(1.09–1.59)	0	.	.	0	.	.	**0.0070**
Colorectum	Colorectum	4080	***1.68*** ^5^	(1.62–1.74)	282	***2.63***	(2.29–3.01)	18	***3.73***	(2.19–6.36)	**<0.0001**
Pancreas	Colorectum	573	**1.13**	(1.02–1.25)	30	1.31	(0.85–2.01)	1	0.87	(0.08–9.18)	**0.0141**
Colorectum	Pancreas	811	***1.16***	(1.08–1.25)	12	1.50	(0.84–2.68)	0	.	.	**<0.0001**
Lung	Colorectum	2023	1.06	(0.99–1.14)	91	1.06	(0.77–1.45)	2	0.48	(0.06–3.97)	0.0890
Colorectum	Lung	1827	**1.07**	(1.01–1.12)	66	1.11	(0.85–1.46)	3	1.09	(0.31–3.87)	**0.0154**
Breast	Colorectum	6090	1.05	(1.00–1.10)	254	1.05	(0.84–1.31)	8	0.78	(0.23–2.72)	**0.0388**
Colorectum	Breast	3051	1.03	(0.99–1.08)	170	1.08	(0.92–1.28)	3	0.34	(0.10–1.17)	0.1068
Endometrium	Colorectum	898	1.03	(0.93–1.15)	50	1.26	(0.82–1.96)	2	1.10	(0.12–9.76)	0.3570
Colorectum	Endometrium	751	**1.10**	(1.01–1.21)	15	1.80	(0.95–3.41)	0	.	.	**0.0250**
Ovary	Colorectum	771	1.06	(0.99–1.14)	32	1.04	(0.74–1.46)	2	1.44	(0.37–5.57)	0.1083
Colorectum	Ovary	619	1.08	(1.00–1.17)	6	1.09	(0.48–2.48)	0	.	.	0.0611
Prostate	Colorectum	5147	1.02	(0.99–1.05)	209	0.94	(0.80–1.10)	8	0.89	(0.39–2.01)	0.4966
Colorectum	Prostate	3608	1.04	(1.00–1.08)	238	1.08	(0.94–1.24)	13	0.57	(0.32–1.02)	0.0534
Melanoma	Colorectum	2101	1.05	(1.00–1.11)	104	**1.33**	(1.06–1.67)	3	1.00	(0.26–3.84)	**0.0096**
Colorectum	Melanoma	782	1.04	(0.96–1.13)	27	**1.56**	(1.01–2.40)	2	3.70	(0.76–18.05)	0.1267
Eye	Colorectum	127	1.19	(1.00–1.42)	8	1.94	(1.00–3.77)	1	5.87	(0.90–38.35)	**0.0151**
Colorectum	Eye	73	0.97	(0.77–1.21)	0	.	.	0	.	.	0.7708
Nervous system	Colorectum	1454	**1.08**	(1.02–1.14)	57	1.10	(0.83–1.45)	5	2.35	(0.92–6.01)	**0.0089**
Colorectum	Nervous system	815	**1.14**	(1.05–1.24)	6	0.60	(0.23–1.55)	1	4.55	(0.44–47.13)	**0.0101**
Thyroid gland	Colorectum	410	**1.15**	(1.03–1.28)	18	1.33	(0.82–2.16)	0	.	.	**0.0064**
Colorectum	Thyroid gland	214	1.07	(0.94–1.22)	4	**3.77**	(1.46–9.71)	0	.	.	0.1845
Myeloma	Colorectum	370	**1.17**	(1.05–1.30)	20	1.44	(0.93–2.24)	1	1.64	(0.23–11.71)	**0.0018**
Colorectum	Myeloma	397	1.06	(0.96–1.17)	3	1.00	(0.31–3.21)	0	.	.	0.2834
CUP^7^	Colorectum	699	1.00	(0.87–1.15)	45	1.44	(0.84–2.49)	2	1.28	(0.10–16.87)	0.6307
Colorectum	CUP	950	**1.15**	(1.05–1.26)	13	1.58	(0.74–3.37)	0	.	.	**0.0022**

^1^RR = relative risk.

^2^CI = confidence interval.

^3^Bold type denotes significantly increased RR at the two-sided 5% level.

^4^Bold and underlined value denotes significantly increased RR at the two-sided 1% level.

^5^Bold, underlined and Italics wvalue denotes significantly increased RR at the two-sided 0.1% level.

^6^Bold type denotes that trend test was statistically significant.

^7^CUP = cancer of unknown primary.

## Discussion

The present study has unsurpassed statistical power to tell which cancers are associated with CRC but multiple comparisons are an unavoidable concern in exploratory studies of the present kind. The present design assumed that for a true familial association more than a single analysis should be positive and RRs should optimally show a ‘dose-response’, i.e., increase by the number of affected probands, beautifully shown for concordant CRC. Endometrial, small intestinal and thyroid cancers showed 3 significant RRs of which some were at 1% or higher confidence level, pancreatic and nervous system cancers showed 2 increased RR of which one was highly significant (<0.1%), and ovarian and eye cancers showed 2 RRs with nominal significance. Lung cancer, myeloma and CUP showed a single significant RR with a 1% confidence level. The remaining cancers showed a single nominal significance; however melanoma showed two associations when potential HNPCC families were removed. Stomach cancer was not association with CRC but showed one positive trend test. There are formal methods of adjustment for multiple comparisons, such as the Bonferroni correction. However, this correction is not very suitable for the present data with several test units of finite number of samples. We would consider two independent significant associations persuasive, as was the case for ovarian cancers which is known to be a common manifestation in HNPCC^8, 14^.

One of the aims of the study was to assess if familial associations could be found outside known CRC related cancer syndromes. The attempt to remove HNPCC families could of course not be fully effective (see next paragraph), for example because of small families, but it showed decreases in RRs and significance levels, particularly for endometrial and ovarian cancers. However, RRs remained essentially unchanged for some cancer types considered HNPCC related, such as small intestinal, pancreatic and central nervous system cancers, leaving the option that the associations were not driven by HNPCC. Thus, for endometrial and ovarian, much of the association with CRC seemed to relate to HNPCC while for the other tumor types the genetics could be more complex.

The present study was nation-wide and we had no access to genetic data which would ethically very sensitive. However, to put the scope of our study in perspective, a recent study reported that 369 Lynch syndrome families were identified in Sweden but no information was given on how many persons were tested for mutations^15^. The authors estimated that no more than one-quarter of the mutation carriers had been identified in Sweden which may imply that some 1500 Lynch syndrome families would exist. Thus our exclusion of 16,160 potential HNPCC families probably included a good proportion of Swedish Lynch syndrome families. However, similar exclusion of families carrying *APC*, *MUTYH* or *POLE and POLD1* mutations because known cancers other than CRC are rare or unknown in these syndrome, and exclusion based on CRC would have defeated the purpose.

The associations of CRC with melanoma, thyroid and eye cancers appeared not to be related to known syndromes. Papillary thyroid cancer, the most common of thyroid cancers, is known to manifest in FAP but CRCs in this syndrome are early onset while the present CRCs associated with thyroid cancer were diagnosed at somewhat higher age than CRCs overall^16^. In a recent study on familial and multiple melanomas we found an RR of 2.63 for CRC in families of at least 2 probands with melanoma and at least one with multiple melanomas^17^. Cutaneous lesions were associated with *MUTYH* mutations but no melanomas were reported nor are such mutations regarded as melanoma predisposing genes^18, 19^. For eye cancer melanoma is the most common histology but RRs did not reach statistical significance for association with CRC. Cutaneous and ocular melanomas are known to share familial risks and *BAP1* gene mutations predispose to both cancers^17, 18, 20^.

In summary, applying the novel approach of testing familial risks in families with increasing numbers of discordant cancers we found persuasive evidence that at least melanoma, thyroid and eye cancers were related to CRC outside known CRC-related syndromes. A number of other cancers associated with CRC in single analyses and independent studies are required to assess the relevance of such findings.

## Methods

Terms HNPCC and Lynch syndrome were initially used interchangeably until Jass defined Lynch syndrome as a disease with a proven mismatch repair gene mutation while HNPCC was a clinical definition based on e.g., the Amsterdam criteria^21^. It is well known that there are HNPCC families lacking mismatch repair gene defects^22, 23^. As our analyses were based entirely on clinical data we used the term HNPCC.

Swedish Family-Cancer Database was created by combining the Multi-Generation Register, national Cancer Registry (started in 1958), and census data. The Database includes all Swedish people born after 1932 (offspring generation) and their biological parents (parental generation). The Database was updated in 2015 containing 15.7 million individuals among which 1.8 million had a cancer diagnosis by the end of 2012.

The 3-digital codes of the 7th revision of the International Classification of Diseases (ICD-7) were used to identify the 35 most common primary cancers and cancer of unknown primary (CUP). For identification of HNPCC families, information on multiple primary cancers was used^13^. If at least one family member in a nuclear family had a double primary consisting of CRC and any of the following cancers: endometrium, ovary, small intestine, pancreas, brain, liver, kidney and bladder, the family was regarded as a likely HNPCC family. For liver and kidney cancers the exclusion may be excessive because only rare hepatobiliary tract cancer and cancer of renal pelvis are considered part of HNPCC^14^. On the other hand, stomach cancers were not used as an exclusion criterion although it may also be included in HNPCC^9^. The order of multiple primaries was not crucial but CRC was required to be present.

The follow-up for cancer in the offspring generation was started from the beginning of 1958, the birth year, or the immigration year, whichever came latest. Follow-up was terminated when a person was diagnosed with cancer, emigrated or died, or at the end of 2012, whichever came first. The number of FDRs (parents and/or siblings) who were affected with cancer was considered as a family history. Incidence rates could be obtained by counting cancer cases and person-years according to family history.

RRs, calculated for the offspring generation, were used as a measure of familial risk by comparing incidence rates for persons with affected relatives (referred to as probands) to incidence rates for those whose probands had no cancer. In the two-way comparison, firstly, RR for cancer X was calculated when family history was CRC, and then in the reverse order RR for CRC was calculated when family history was cancer X. For parents and offspring (large majority of familial cases) these comparisons are independent but for siblings the pairs of cases are the same. Significant results in two-way analyses provide support for a true association but a lacking two-way association is no strong evidence against an association because age distributions and case numbers may differ between two-way analyses.

Poisson regression modeling was employed to estimate RRs and corresponding confidence intervals (CI) for 5%, 1% and 0.1% significance levels. Trend tests were performed by modeling the number of familial cancers as a continuous covariate. Potential confounders, including sex, age group (5-year bands), period (5-year bands), socioeconomic status (blue collar worker, white collar worker, farmer, private, professional, or other/unspecified), residential area (large cities, South Sweden, North Sweden, or unspecified) were added to the model as covariates. SAS version 9.4 was used to perform the statistical analysis.

### Ethical statement

The study was approved by the Ethical Committee of Lund University and the study was conducted in accordance with the approved guidelines.
